# A Touchscreen Motivation Assessment Evaluated in Huntington's Disease Patients and R6/1 Model Mice

**DOI:** 10.3389/fneur.2019.00858

**Published:** 2019-08-09

**Authors:** Christopher J. Heath, Claire O'Callaghan, Sarah L. Mason, Benjamin U. Phillips, Lisa M. Saksida, Trevor W. Robbins, Roger A. Barker, Timothy J. Bussey, Barbara J. Sahakian

**Affiliations:** ^1^Department of Psychology, Behavioural and Clinical Neuroscience Institute, University of Cambridge, Cambridge, United Kingdom; ^2^School of Life, Health and Chemical Sciences, The Open University, Milton Keynes, United Kingdom; ^3^Brain and Mind Centre, University of Sydney, Sydney, NSW, Australia; ^4^John van Geest Centre for Brain Repair, Addenbrooke's Hospital, University of Cambridge School of Clinical Medicine, Cambridge, United Kingdom; ^5^Department of Physiology and Pharmacology, Schulich School of Medicine and Dentistry, University of Western Ontario, London, ON, Canada; ^6^Department of Psychiatry, University of Cambridge School of Clinical Medicine, Cambridge, United Kingdom

**Keywords:** Huntington's disease, apathy, touchscreen, translational, motivation, progressive ratio

## Abstract

Apathy is pervasive across many neuropsychiatric disorders but is poorly characterized mechanistically, so targeted therapeutic interventions remain elusive. A key impediment has been the lack of validated assessment tools to facilitate translation of promising findings between preclinical disease models and patients. Apathy is a common symptom in Huntington's disease. Due to its established genetic basis and the availability of defined animal models, this disease offers a robust translational framework for linking motivated behavior with underlying neurobiology and an ideal context in which to evaluate a quantitative, translational apathy assessment method. In this study we therefore aimed to demonstrate the validity of using touchscreen-delivered progressive ratio tasks to mirror apathy assessment in Huntington's disease patients and a representative mouse model. To do this we evaluated Huntington's disease patients (*n* = 23) and age-matched healthy controls (*n* = 20), and male R6/1 mice (*n* = 23) and wildtype controls (*n* = 29) for apathy-like behavior using touchscreen-delivered progressive ratio tasks. The primary outcome measure of the assessment was breakpoint, defined as the highest number of touchscreen responses emitted before task engagement ceased. Patients and R6/1 mice were both found to exhibit significantly reduced breakpoints relative to their respective control groups, consistent with apathy-like behavior. This performance was also not associated with motoric differences in either species. These data demonstrate the utility of touchscreen-delivered progressive ratio tasks in detecting clinically relevant motivational deficits in Huntington's disease. This approach may offer a platform from which clinically relevant mechanistic insights concerning motivation symptoms can be derived and provide an effective route for translation of promising preclinical findings into viable therapeutic interventions.

## Introduction

Apathy is a multidimensional construct that encompasses a wide range of clinical features, including reductions in goal-directed behavior, cognitive activity, and emotional expression (1–4). It is a prominent feature of many neurodegenerative and neuropsychiatric disorders including Alzheimer's disease, Parkinson's disease, Huntington's disease (HD), and schizophrenia (5–8).

Huntington's disease is classically conceptualized with a triadic presentation of motoric, cognitive, and neuropsychiatric symptoms (9). Apathy is one of the earliest neuropsychiatric symptoms (10) with prevalence rates of 34–76% across the disease course (11–13) and can present in advance of the motoric symptoms associated with the disease (14). Unlike other neuropsychiatric features of HD, apathy progressively worsens as the disease advances (10, 15) and is associated with a broader decline in other cognitive domains and daily functioning (16).

Despite its prevalence and impact on quality of life for both patients and caregivers, apathy remains a difficult symptom to treat effectively in HD, with no successful HD apathy treatment trial reported to date (17). This is partly due to the often mixed neuropsychiatric presentation of these patients and concerns around polypharmacy particularly related to medication interactions and side-effects leading to exacerbation of other neuropsychiatric symptoms (14, 18). Beyond these clinical practice-related concerns, effective treatment of apathy is also considered challenging as it is linked to a range of etiologies involving multiple neural systems (19) such that underlying causal mechanisms remain unknown. Indeed, distinct from other neuropsychiatric symptoms associated with HD, apathy has not yet been associated with physiological biomarkers such as plasma cytokines (20) or white matter fractional anisotropy (21), potentially indicating a multi-factorial pathophysiological cascade, though a recent study did report an association with changes in cortical CB_1_R expression (22). Furthermore, objective measures for apathy, which are needed to detect and monitor symptom severity and track the effectiveness of interventions, are lacking. Therefore, identification of viable neuropharmacological targets and screening of prospective treatments for efficacy is particularly challenging.

A substantial proportion of apathy-targeted research has utilized subjective questionnaire assessments. Questionnaires are problematic for longitudinal tracking of symptoms and for clinical trials, as they rely on the accuracy of patient self-report or require an observant and unbiased informant, with some indications that the extent of correlation between reports of neuropsychiatric symptomology in HD from patients relative to informants varies as a function of disease state, suggesting issues related to patient insight can impact severity estimates (23, 24). Furthermore, variability in the approach used to evaluate apathy in HD has been suggested as a contributor to the wide prevalence rate estimates for this symptom (14). To advance understanding of apathy in HD and generate efficacious treatment strategies, there is therefore a pressing need to develop more objective motivation assessments that can ideally be used both preclinically and in patients (25).

Touchscreen-delivered motivation assessments could offer a route to achieve this goal (26–29). Performance in touchscreen assessments targeting other cognitive domains in humans (30, 31) and rodents (32–34) are similarly impacted by mutations in a homologous disease related gene (*Dlg2)* (35), and in Alzheimer's disease patients and the 3xTgAD rodent model of the disease (36). Findings derived from these tools therefore have the potential to facilitate rapid identification of relevant neurobiological substrates, pathological mechanisms, and novel therapeutics.

We have capitalized on this approach to assess motivational deficits in HD using analogous touchscreen-delivered progressive ratio (PR) tasks in patients and in a well-characterized transgenic mouse model of this disease. In PR schedules, participants earn a reward following emission of a defined number of responses. The number of responses required for each subsequent reward increases according to a pre-defined sequence. This continues until the participant reaches their breakpoint, which is defined as the number of responses emitted for the last reward successfully earned before responding ceases (37). Breakpoint is taken as an index of the reinforcing effects of a stimulus (38) and is based on an effort-cost computation where reward value is weighed against effort expenditure (39). A PR schedule can therefore measure specific sub-processes of motivated behavior, namely reward sensitivity and sustaining effort. These schedules have been widely used in animals and humans to evaluate the reinforcing effects of drugs and the impact of varying dosages (40, 41). More recently, however, their utility in measuring motivation in neuropsychiatric conditions has been demonstrated in schizophrenia, where breakpoints have been related to clinical amotivation as assessed by behavioral scales (39, 42) and in relevant animal disease models (43, 44).

Here, we utilized a newly developed touchscreen-based PR task to objectively assess motivation in HD patients (31). This task is largely non-verbal and the associated cognitive demands are low, making it suitable for use in a range of clinical conditions. In parallel, we employed the rodent touchscreen PR schedule (45, 46) to evaluate motivation in the R6/1 mouse model. In addition to numerous similarities in paradigm design and implementation, these assessments provide a variety of analogous outcome measures thereby facilitating direct inter-species comparison of performance metrics. Taken together, this study presents an initial characterization of the touchscreen PR schedule as a tool with utility in HD populations and with high translational potential for the assessment of motivation in this disease context.

## Materials and Methods

### Human Participant Case Selection

Huntington's disease patients (*n* = 23) were recruited from the regional NHS Huntington's disease service clinic at the John van Geest Center for Brain Repair, Cambridge, UK. All had received a diagnostic confidence rating of 4 by an accredited HD clinician. Of these, 20 were receiving HD treatment (either monotherapy with olanzapine, amantadine, tetrabenazine or risperidone; or combinations of these) and 13 were also treated for affective problems (with selective serotonin reuptake inhibitors or benzodiazepine monotherapy, or a combination thereof).

Patients were evaluated using the UHDRS and scores for total functional capacity and functional activity scales as well as total motor scores were recorded. The Addenbrooke's Cognitive Examination-Revised [ACE-R; (47)] was administered as a measure of general cognition and the Cambridge Behavioral Inventory-Revised [CBI-R; (48)] was used to evaluate behavioral symptoms.

Age-matched controls (*n* = 20) were recruited from a volunteer panel at the Behavioral and Clinical Neuroscience Institute, University of Cambridge, UK. Exclusion criteria for controls were presence of known neurological or psychiatric disorders, use of psychoactive medications or significant head injury. Older controls (over 50 years) were screened for cognitive impairment using the ACE-R with the established cut-off of 88 or above (47).

The human arm of the study was approved by the local Research Ethics Committee (reference number 09/H0308/2) and Cambridge University Hospitals NHS Foundation Trust Research and Development Department. All participants provided informed consent in accordance with the Declaration of Helsinki. See Table 1 for demographic details and clinical characteristics.

**Table 1:** Demographic and clinical characteristics of human participants.

**Demographic/clinical characteristic**	**HD**	**Control**	***p*-value**
*N*	23	20	–
Sex (M:F)	13:10	10:10	–
Age	53.6 (25–76; 14.6)	52.2 (20–81; 20.2)	n.s.
MMSE (max. 30)	26.0 (19–30; 3.0)	29.7 (28–30; .63)	***
ACE-R (max. 100)	77.6 (56–93; 12.9)	97.2 (96–99; 1.7)	***
CBI-R (max. 180)	53.1 (13–114; 23.7)		
Total motor score	28.0 (4–50; 13.9)		
Functional activity	18.0 (11–25; 4.3)		
Total functional capacity	8.7 (3–25; 4.8)		

Mean (range; standard deviation). n.s., non-significant;

*** *p < 0.001. MMSE, Mini-Mental State Examination; ACE-R, Addenbrooke's Cognitive Examination-Revised; CBI-R, Cambridge Behavioral Inventory-Revised*.

### Apathy Questionnaire Assessment

An informant-rated version of the Apathy Evaluation Scale (AES) (49) was employed as a clinical apathy measure in the HD patients. The AES assesses behavioral and psychological manifestations of apathy over the past 4 weeks and item frequency is scored on a four-point scale. AES scores are reported as a percentage, with higher values indicating increased levels of apathy.

Controls completed a self-rated apathy questionnaire (LARS-e). Based on the Lille Apathy Rating Scale (LARS) (50), the LARS-e is modified and extended to be sensitive to motivational variations in healthy populations (51). Items are rated on a five-point scale. LARS-e scores are reported as their reciprocal percentage, with higher scores indicating increased levels of apathy.

### Human Progressive Ratio Task

This task is part of the EMOTICOM battery (31) and is administered on a touchscreen laptop (Dell XT3). A maximum of 437 trials are possible in this task, each being self-paced with participants required to press a “Next” button to begin each trial. The task has three consecutive trial blocks associated with a progressively smaller reward value (£1, 20p, and 4p).

In each trial, four red squares are presented and participants are instructed to select the square that differs in size to the other three. Participants initially need to complete 4 selections to receive a reward, with this doubling to 8, 16, and 32 responses. Participants are informed that they can stop performing the task at any point, but they must then sit facing the screen for any remaining session time. The task is stopped by pressing a “Quit” button, which remains available throughout. Participants were not rewarded with money in this study as performance-based reimbursement was not permitted by the ethics committee, but they were instructed to engage as if real money was at stake.

Breakpoint was defined as the number of trials completed before quitting the task. Other outcome measures included the post reinforcement pause, defined as the average time taken to initiate the next trial following a rewarded trial, and the running rate, defined as the number of responses emitted per second over the task correcting for the post reinforcement pause (i.e., responses/total time in seconds minus post reinforcement pauses).

### Animals

Male R6/1 mice (52) on the B6CBAF1/J background bred at the University of Cambridge were utilized in this study. R6/1 (*n* = 23; mean CAG repeat length = 120 ± 0.29) and wildtype (WT) littermate controls (*n* = 29) were moved from the breeding facility at 6 weeks of age and housed in the behavioral assessment facility in mixed genotype groups in a humidity- and temperature-controlled housing room with a 12 h light-dark cycle (lights off: 0700). Mice were left undisturbed except for normal husbandry for 7 days following transfer to allow facility acclimatization. Cages were changed once weekly and drinking water bottles twice weekly. All animals experienced single daily behavioral training sessions 5–7 days per week with experimenters blind to genotype. All procedures were conducted during the dark phase of the cycle, reviewed and approved by the University of Cambridge AWERB and performed in accordance with the United Kingdom Animals (Scientific Procedures) Act (1986) and the United Kingdom Animals (Scientific Procedures) Act (1986) Amendment Regulations 2012.

### Food Restriction and Reward Habituation

From 7 weeks of age, animals were regularly handled to habituate them to the experimenters and weighed daily to establish stable free-feeding weights. Food restriction to ~85–90% of free-feeding weight consisted of providing limited amounts of standard laboratory chow (RM 3; Special Diet Services, Essex, UK) daily.

Animals were habituated to the liquid reward used in the PR assessment (Yazoo Strawberry UHT milkshake; FrieslandCampina UK, Horsham, UK), by placing a sample in a small bowl in each cage for two consecutive days immediately prior to behavioral training. The liquid reward sample was provided coincident with chow pellet delivery to minimize neophobia (32).

### Apparatus

Behavioral assessments were conducted in standard Bussey-Saksida mouse touchscreen chambers (Campden Instruments Ltd., Loughborough, UK) as described in detail previously (32–34). Briefly, these chambers consist of a behavioral arena with a perforated stainless steel floor and trapezoidal black plastic walls which open on to a touchscreen (12.1 inch; resolution 800 × 600). In these chambers, an array of infrared (IR) photo-detection beams are projected immediately above the surface of the touchscreen so that animals do not have to exert any mechanical pressure to successfully register a response. A reward collection magazine is located on the wall opposite the touchscreen. This contains an LED which is illuminated upon reward delivery.

Each arena is housed within a dense fibreboard sound attenuating chamber equipped with an LED house light and a fan to provide ventilation and mask background noise. Animals are observed through overhead IR-sensitive cameras and IR emitters sited along the length of the arena. The chambers are also equipped with IR photo-detection beams which run across the floor of the arena (rear beam = 3 cm from magazine port and front beam = 6 cm from screen) to monitor horizontal locomotor activity independently of task-specific locomotor proxy measures. A tone generator and speaker are also located directly above the behavioral arena.

For this study the standard “5-choice” mask (Campden Instruments Ltd.) was placed in front of the touchscreen (45, 46). This mask contains a row of 5 square apertures measuring 4 × 4 cm, each spaced 1 cm apart and positioned 1.5 cm above the floor of the arena. The mask was used to guide responding and minimize unintended screen contact by the mice. The central response aperture was the only location in which stimuli were presented in this study.

### Touchscreen Behavioral Chamber Training

Mice were first habituated to the chambers and then underwent initial operant training to associate stimulus offset with reward delivery in the magazine unit positioned opposite the touchscreen. A fixed ratio (FR) schedule requiring an invariant number of touchscreen responses to yield reward delivery was then introduced. Reward volume was set at 20 μL throughout.

#### Behavioral Chamber Habituation

Mice were habituated to the chambers with a single 20 min session in which the animals were placed in the chambers and locomotor beam breaks, magazine entries, and screen touches were recorded. There were no programmed consequences for any behavioral response. To facilitate habituation, 200 μL of liquid reward was delivered to the magazine at the start of the session.

#### Initial Operant Training

Initial operant training consisted of one 60 min session in which animals were trained to associate stimulus offset with reward delivery. This session consisted of 30 trials in which the target stimulus (a white square) was presented in the central touchscreen response aperture. The stimulus was displayed for 30 s and upon stimulus offset a tone (3 kHz, 1,000 ms) was presented, the magazine illuminated and liquid reward (20 μL) delivered. The magazine remained illuminated until reward collection, at which point a 5 s inter-trial interval (ITI) was enforced. Upon completion of the ITI the next trial began with stimulus display. If during presentation the animal touched the target stimulus, it was immediately removed from the screen, the tone was presented, the magazine was illuminated and a triple volume of liquid reward (60 μL) was delivered. Mice were required to collect 30 rewards in a single session before progression to the next stage of training.

#### Fixed Ratio Training

Animals were then introduced to the fixed ratio (FR) contingency as described previously (46). Initially this consisted of a single session of FR 1 training in which the white square stimulus was presented in the central touchscreen response aperture until the animal made a response in that location. Responding resulted in stimulus removal, tone presentation, magazine illumination and reward delivery (20 μL). Animals were given 12 trials of this contingency with a maximum session time of 60 min and a 4.5 s ITI.

The day after successful completion of 12 FR 1 trials, animals progressed to the FR 2 schedule. This was identical to the FR 1 schedule except that two screen touches were required for each reward delivery. In this schedule, the first screen touch resulted in brief (500 ms) removal of the target stimulus and delivery of a “chirp” tone (3 kHz, 10 ms) to indicate that a response had been successfully registered. The second screen touch had the same consequences as in the FR 1 schedule. Animals were required to complete 6 FR 2 trials (thereby emitting the same total number of responses as in the FR 1 schedule) to successfully complete this training phase. Upon completion of FR 2 training, animals were moved to the FR 3 schedule in which three responses were required for each reward delivery. The other parameters of this program were identical to the FR 2 schedule, except that animals were required to complete 4 trials under this contingency to standardize the total number of responses emitted in each FR training phase to 12.

Upon completion of FR contingency training and to ensure performance stability, animals were given a further seven consecutive sessions of the FR 3 schedule. The parameters of these sessions were identical to those used previously, except that the maximum number of trials permitted was increased to 8 to ensure responding was sustainable. Upon completion of these sessions, animals were rested for up to 3 days to allow any mice delayed in training to successfully reach the necessary performance criteria. All animals were then given two further 8 trial sessions of the FR 3 schedule and permitted to progress to the PR assessment upon completion of 8 trials in a single session. This training protocol resulted in one R6/1 animal being delayed by a single session.

### Progressive Ratio Assessment

PR assessment was conducted as previously reported (45, 46). The schedule parameters used were identical to those established in the FR contingency training, except that upon successful completion of a trial the subsequent response requirement for reward delivery was increased on a linear +2 basis (i.e., a PR 2 schedule requiring 1, 3, 5, 7, 9 responses, etc.). To minimize differential reward exposure, a limit of 45 trials was applied in these sessions. An inactivity timeout was also enforced that specified if an animal did not make a screen response after 5 min of stimulus display or make a magazine entry within 5 min of reward delivery, the behavioral session ended and the mouse was removed from the chamber.

Breakpoint was operationally defined as the number of responses emitted to obtain the last reward before inactivity time out or session completion. The total number of screen responses was also analyzed to enable assessment of responses emitted beyond those required for the last successfully completed trial. Response rate was calculated via conversion of the inter-reinforcer interval to rate per trial (53–55). Rates for individual subjects were fitted with a negative exponential function *y* = *a*^−*bx*^ where y represents response rate, a represents y intercept, –b represents decay, and x represents trials (44, 55, 56). The intercept (a) and decay (–b) parameters were then extracted and tested for between-group statistical significance.

Animals were given three consecutive PR 2 sessions and the mean performance of each animal was used for analysis. PR data were collected when the animals were 11 weeks of age to ensure that the well-characterized age-dependent impairment in motor output in this strain (52) did not confound any observed differences in task performance.

### Locomotor Activity Assessment

The locomotor capabilities of the animals used in this study were also evaluated on the same day as the final PR assessment session. For this assessment the touchscreen chambers were converted to the “autoshaping” configuration (32, 57) to provide a distinct context for the animals. In this configuration the magazine was moved to a central location directly in front of the touchscreen and the five aperture mask was replaced with a two aperture version with one aperture on either side of the magazine. To further enhance the contextual difference, the houselight was illuminated during this assessment. In this evaluation, mice were placed in the chambers for 10 min and in that time the number of locomotor activity beam breaks, magazine entries and screen touches was recorded. This provides a proxy assessment of locomotor activity based on the exploration of a relatively novel environment and should not be confounded by factors related to the PR assessment. It therefore provides a reliable evaluation of motoric capabilities.

### Statistical Analyses

Analyses were conducted using the open-source statistical environment R (58). Variables were checked for normality using the Shapiro-Wilk test. For the human participants, data were compared using Mann-Whitney *U*-tests. Cohen's d effect size was computed for all comparisons. Correlations were analyzed using Spearman rank coefficients. For the rodent data, Welch's two sample *t*-tests were applied. Unless otherwise indicated, data are presented as mean ± standard error of the mean. The Bonferroni correction for multiple comparisons was applied as necessary, and all analyses are two-tailed at an α level of 0.05.

## Results

### Huntington's Disease Patients Show Reduced Motivation on the Touchscreen PR Task

**Figure 1:**
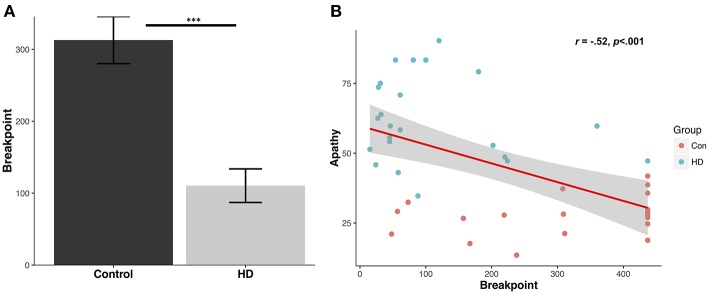
Human progressive ratio breakpoint and correlation with questionnaire apathy scores. **(A)** Average breakpoint for controls and HD patients; ^***^p < 0.001. **(B)** Levels of questionnaire-determined apathy for controls and patients (LARS-e and AES) correlate with progressive ratio breakpoint.

Patients demonstrated a significantly reduced breakpoint value relative to controls (Control mean: 312.9 ± 146.4 (SD); Patient mean: 110.4 ± 111.8 (SD); *U* = 394.0, *p* < 0.001, *d* = 1.6) (Figure 1A). Running rate was also significantly reduced in the patient group (Control: 0.5 ± 0.1 responses per second; Patient: 0.2 ± 0.1 responses per second; *U* = 17.0, *p* < 0.001, *d* = 2.2). Post reinforcement pause was significantly elevated in patients (Control: 0.95 ± 0.42 s; Patient: 2.6 ± 1.2 s; *U* = 56.0, *p* < 0.001, *d* = 1.4).

Breakpoints in the patient group were highly correlated with their total functional capacity, a global indicator of functional decline in everyday activities with lower scores indicating more severe impairment (*r* = 0.45, *p* < 0.05), but not with their UHDRS total motor scores (*r* = −0.37, *p* = 0.09). Furthermore, neither total functional capacity or UHDRS total motor scores were significantly correlated with the post reinforcement pause or running rate (*p* values > 0.17).

### Questionnaire-Derived Apathy Levels Correlate With PR Performance

The mean control group apathy score using LARS-e was 27.9 ± 7.2%. The mean apathy score in the patient group using AES was 61.9 ± 15.3%. A significant negative correlation was detected between apathy questionnaire score (higher indicating greater apathy) and PR breakpoint value across the groups (*r* = −0.52, *p* < 0.001) (Figure 1B), suggesting that higher levels of everyday apathy were associated with reduced motivation in the PR task.

### R6/1 Transgenic Mice Exhibit Reduced Motivation in the Touchscreen PR Assessment at 11 Weeks of Age

**Figure 2:**
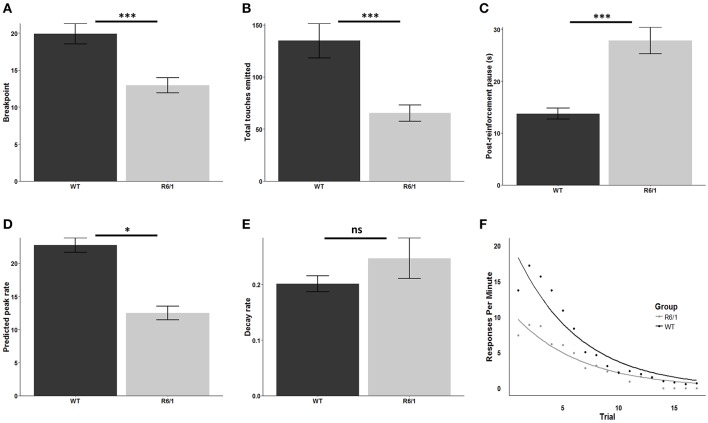
Progressive ratio performance in R6/1 and wildtype mice. Rodent progressive ratio performance: **(A)** breakpoint; **(B)** total screen touches; **(C)** post-reinforcement pause; **(D)** predicted peak performance; and **(E,F)** decay rate. WT, wildtype; R6/1, HD model; ^*^p < 0.05; ^***^p < 0.001; ns, non-significant.

R6/1 mice were significantly less motivated to obtain the palatable strawberry milkshake reward relative to wildtype littermates as assessed by breakpoint [mean WT breakpoint: 19.92 ± 1.38; mean R6/1 breakpoint: 12.94 ± 1.00; *t*_(48.162)_ = 4.0879; *p* < 0.001; *d* = 1.09; Figure 2A] and total screen touches [mean WT total touches: 134.78 ± 16.63; mean R6/1 total touches: 65.28 ± 7.94; *t*_(39.603)_ = 3.7714; *p* < 0.001; *d* = 0.97; Figure 2B]. Post reinforcement pause was also significantly elevated in the R6/1 animals relative to WT [mean WT PRP: 13.73 ± 1.07; mean R6/1 PRP: 27.84 ± 2.56; *t*_(29.606)_ = −5.0861; *p* < 0.001; *d* = −1.53; Figure 2C].

### R6/1 Transgenic Mice Exhibit Reduced Maximal Operant Output in Touchscreen PR

Comparison of the estimated peak PR response rate, which represents the projected maximum possible rate of touchscreen responding, indicated a significant reduction in R6/1 animals relative to wildtype littermates, consistent with a lower intrinsic motivational baseline [WT: 22.76 ± 1.08; R6/1: 12.45 ± 1.03; *t*_(45.687)_ = 6.892; *p* < 0.001; *d* = 1.96; Figure 2D]. In contrast, comparison of the response decay rate, which represents the decline in the rate of touchscreen responding across the PR session, revealed no significant effect of genotype [WT: 0.20 ± 0.014; R6/1: 0.25 ± 0.037; *t*_(26.075)_ = −1.159; *p* = 0.2568; Figures 2E,F].

### Touchscreen PR Performance Was Not Confounded by Generalized Locomotor Disturbance or Body Weight Changes in R6/1 Mice

R6/1 touchscreen PR performance was assessed at 11 weeks of age which is ~4 weeks prior to the onset of any reported motor deficits in this HD model (52). To confirm the absence of any potentially confounding motoric deficits, the locomotor activity of the animals was assessed in a novel configuration of the touchscreen chamber.

No significant differences were detected between R6/1 and WT littermates across several measures including the number of infrared beam breaks across the chamber floor adjacent to the reward magazine [WT: 26.69 ± 2.27; R6/1: 26.68 ± 3.01; *t*_(41.715)_ = −0.0569; *p* = 0.9549], the number of infrared beam breaks across the floor of the chamber adjacent to the left side of the touchscreen [WT: 80.31 ± 5.28; R6/1: 94.05 ± 9.55; *t*_(33.475)_ = 1.2585; *p* = 0.2169], the number of infrared beam breaks across the floor of the chamber adjacent to the right side of the touchscreen [WT: 79.93 ± 7.50; R6/1: 75.82 ± 7.29; *t*_(48.358)_ = −0.39318; *p* = 0.6959], the number of ambulatory transitions from the touchscreen to the magazine [WT: 16.28 ± 1.19; R6/1: 14.32 ± 1.33; *t*_(45.944)_ = −1.0941; *p* = 0.2796], the number of touches recorded on the left side of the touchscreen [WT: 55.14 ± 3.52; R6/1: 51.77 ± 3.95; *t*_(45.899)_ = −0.63641; *p* = 0.5277] and the number of touches recorded on the right side of the touchscreen [WT: 49.24 ± 3.68; R6/1: 56.77 ± 5.82; *t*_(36.74)_ = 1.0932; *p* = 0.2814]. A numerical but non-significant increase in the number of magazine entries made by the R6/1 animals was detected [WT: 9.86 ± 1.35; R6/1: 19.82 ± 4.90; *t*_(24.2)_ = 1.9605; *p* = 0.06155].

Age-dependent changes in body weight have also been reported in the R6/1 mice (52) and as this PR assessment was based on the collection of a palatable strawberry milkshake reward it was important to exclude this factor as a contributor to the observed performance impairment. Comparison of body weight during the PR assessment, when expressed as the mean (WT: 22.95 ± 0.33g; R6/1: 22.54 ± 0.42 g; *p* = 0.4459) or as the mean percentage of free feeding weight (WT: 89.03 ± 0.90; R6/1: 87.64 ± 0.67; *p* = 0.2213), revealed no significant differences between the genotypes.

## Discussion

In this study, we have demonstrated the potential of a fully quantitative, touchscreen-delivered motivational assessment to detect apathy-like behavior in HD patients and a well-characterized rodent disease model.

Specifically, we have shown that manifest HD patients exhibit lower breakpoints relative to healthy controls in a touchscreen PR assessment. Critically, task performance was related to everyday levels of apathy as determined by behavioral questionnaires, but not motor scores. Using an analogous rodent touchscreen PR assessment, we found that the R6/1 transgenic HD mouse model exhibited lower breakpoints relative to wildtype littermates in the absence of any generalized locomotor disturbance.

Our finding of increased apathy in patients on an established questionnaire is consistent with previous studies (7, 10, 13). The extent of apathy on such scales has been related to reduced quality of life (59), highlighting the importance of apathy as a therapeutic target. The correlation between PR breakpoint and the AES we identified suggests that touchscreen PR may provide an effective approach to objectively measure apathy in HD. On the basis that apathy severity can be quantitated using this approach, touchscreen PR may ultimately be a useful tool to objectively assess motivation deficits in future studies that can inform the mechanistic basis of apathy and serve as an outcome measure in interventional studies.

Our patient group comprised a convenience sample of individuals attending our HD clinic, and therefore represents a wide range of disease stages. The degree of patient engagement with the task that we observed here highlights the potential for the touchscreen PR platform to be used for longitudinal motivational assessment of patients across the whole disease course. It will be important in future work to further validate the task in cohorts at discrete disease stages to determine its sensitivity to detect motivational deficits, particularly in the prodromal phase when such deficits may be subtle. Following such prodromal patients longitudinally using the touchscreen PR platform represents a further important future validation study, as does appropriate evaluation of test-retest reliability.

Given that our patient cohort was a convenience sample, a wide range of different pharmacotherapies was represented. That touchscreen PR was able to detect a motivational impairment emphasizes the point that current HD treatment approaches are unable to effectively ameliorate the apathy associated with this condition (10, 14, 17). However, the pharmacotherapeutic heterogeneity in the sample, coupled with concerns around medications used in HD potentially exacerbating neuropsychiatric symptom severity (14) means we cannot rule out the possibility that the magnitude of apathy detected in some of the patients here may have been enhanced by their medication profile. In order to fully disentangle the relative contributions of medication and pathophysiology to the level of motivation quantitated by touchscreen PR in HD, assessment of cohorts with uniform pharmacotherapeutic profiles or cohorts in which medication is temporarily suspended will be necessary in future studies.

The impaired PR performance exhibited by the R6/1 mice, ahead of any motor deficits, further increases the face and construct validity of these animals as a means to explore HD motivational deficits (60). Impairments in motivation to earn reward have been reported in other HD rodent models including the BAC HD, z_Q175 KI and *Hdh* mouse strains (61–65) and the BACHD rat (66–68). Given that the R6/1 mouse is widely studied, particularly with respect to novel therapeutics, it offers a powerful platform, in combination with the PR task, to deliver novel insights into the pathophysiological mechanisms underlying this early onset HD symptom and to evaluate potential therapeutic avenues.

The striking consistency between the human and rodent PR data observed here complements findings in touchscreen studies targeting other cognitive domains involving humans and mice carrying mutations in a schizophrenia-related gene (35, 69) and in Alzheimer's patients and a rodent disease model (26, 36). Our findings establish the utility of this approach in the context of Huntington's disease. As such, touchscreen PR may ultimately facilitate apathy-targeted therapeutic translation in HD by standardizing the assessment and output measures used to evaluate motivation in both patient and model systems.

A challenge of evaluating motivation using effort expenditure tasks in movement disorder patients and corresponding rodent models is the potential for confounding due to generalized motor slowing and motor impairment. While the reduced breakpoint observed in patients here was accompanied by reduced running rate and increased post reinforcement pause, PR task performance was not significantly correlated with clinical assessment scores of motor function. These results suggest that gross motoric deficits are unlikely to account for the observed PR deficits.

Similarly, the R6/1 model is known to develop significant motoric dysfunction (52) which could likewise contribute to PR performance deficits. However, when general locomotor activity was assessed, no significant impairments were observed. Consistent with the patient data, R6/1 mice also exhibited a significantly increased post-reinforcement pause—a parameter that typically is associated with changes in motivational state as opposed to locomotor activity (70, 71). Indeed, given this absence of a motoric impairment in the transgenic animals, the significant reduction in estimated peak response rate in the R6/1 group observed here is therefore consistent with a lower intrinsic motivational baseline in these animals (55). Taken together, these observations indicate that the profound impairment in PR performance detected in the R6/1 animals was not due to motor impairment but rather represents a genuine motivational deficit.

In this study, we have demonstrated the utility of the touchscreen PR paradigm in a representative clinical sample of HD and detected the expression of an apathy-like phenotype in a well-characterized rodent model of the disease. Future studies employing this approach are now required to measure apathy at different disease stages in both clinical and pre-clinical contexts. More broadly, the findings reported here highlight the potential for the touchscreen PR schedule to provide an important platform upon which to investigate the neurobiological underpinnings of disrupted motivation and to evaluate novel interventions intended to ameliorate such disruptions across a range of neurodegenerative and neuropsychiatric disorders. These findings also indicate the capacity for the touchscreen PR schedule to be leveraged for either forward or reverse translational studies in this area.

However, these conclusions should be tempered by noting the assessment of a convenience patient sample here, which represents heterogenous disease state and pharmacotherapeutic administration profiles. Similarly, while this study contributes crucial further evidence of the cross-species translational capabilities of touchscreen delivered cognitive assessments (26, 35, 36, 69) the considerable degree of face validity between the human and non-human touchscreen assessments achieved here does not, in the absence of further validation, guarantee the presence of similar levels of construct and predictive validity. Efforts to evaluate construct and predictive validity are advancing for a range of touchscreen tasks (72, 73) and given the long history of PR schedules in studies of non-human species (37, 74) and the resulting substantial evidence base covering the effects of a range of manipulations on performance, combined with the recent implementation of PR schedules in humans (31, 74), such validation studies for PR are now eminently feasible. Indeed, with these considerations in mind, an important development will therefore be to apply this translational paradigm in a cohort of premanifest individuals who are not yet exhibiting the motor signs of HD. Such a group would more closely reflect the early stage deficits we have shown in the 11 week old R6/1 mice here.

Taken together, we have established the utility of touchscreen PR as an approach for the objective, fully quantitative assessment of motivation in HD. To develop apathy-targeted therapeutics for HD and other neuropsychiatric disorders, it will be necessary to identify the specific sub-processes of motivated behavior that are disrupted as well as the underlying circuitry and neurochemical alterations. It follows that sound translational models in which putative therapeutics can be evaluated will be essential for progress in this area. The cross-species validated touchscreen PR paradigm, as a proxy measure for apathy, has the potential to address these needs and ultimately support improved treatments for this symptom in Huntington's disease and other disorders.

## Data Availability

The mouse raw data supporting the conclusions of this manuscript will be made available by the authors, without undue reservation, to any qualified researcher. The data from human participants cannot be made available as the ethics did not cover open data sharing.

## Author Contributions

CH, CO'C, BP, and SM organized and performed experiments. CH, BP, and CO'C designed and performed statistical analyses which were reviewed by the other authors and wrote the first draft of the manuscript. RB, BS, TR, LS, and TB provided key resources, access to critical research infrastructure and oversight of the project. CH and CO'C made revisions based on the review and critique provided by all the other authors. All the authors contributed to the conception and design of the study. BS and TR are co-inventors of the Human Touchscreen Progressive Ratio Task.

### Conflict of Interest Statement

TR discloses consultancy with Cambridge Cognition, H. Lundbeck A/S, Unilever and Mundipharma and has research grants with H. Lundbeck A/S and Shionogi. BS consults for Cambridge Cognition, PEAK, and Mundipharma. LS and TB consult for Campden Instruments, Ltd. The remaining authors declare that the research was conducted in the absence of any commercial or financial relationships that could be construed as a potential conflict of interest.
